# Dubito Ergo Sum. Pathologies that can Mimic Sepsis

**DOI:** 10.2478/jccm-2022-0011

**Published:** 2022-05-12

**Authors:** Bianca-Liana Grigorescu

**Affiliations:** 1 Department of Pathophysiology, George Emil Palade University of Medicine, Pharmacy, Science, and Technology of Targu-Mures, Targu-Mures Romania

Sepsis is a potentially deadly organ dysfunction caused by a dysregulated host response to infection, with a high mortality rate [1]. Generally, sepsis is acquired in the community, and its development is slow, making diagnosis challenging. Early broad-spectrum antibiotics and effective source management improve prognosis [1, 2].

Sepsis has a huge financial impact on the health-care system; septic patient treatment in the United States alone is projected to cost more than $20 billion per year. The cost in human life is equally high; mortality rates in sepsis and septic shock are believed to be more than 10% and 40%, respectively [3]. Sepsis is one of the most prevalent causes for admission to the intensive care unit (ICU) and the leading cause of mortality in ICUs across the globe [3, 4].

Although bacterial infections are the most prevalent cause, viruses and fungi may also cause infections in patients with co-morbid disorders and immunosuppression. Infections of the lower respiratory tract are the most often seen foci in hospitalized patients, followed by infections of the intra-abdominal, bloodstream, intravascular line, and urine tract [1].

In clinical practice, both the SEPSIS-2 and SEPSIS-3 definitions are now utilized, each with its own language and set of criteria, such as blood pressure and lactate cutoff points. SEPSIS-3 employs sequential organ failure assessment (SOFA) or the rapid version (qSOFA) to define sepsis, and SEPIS-2 criteria continues to rely on SIRS criteria for sepsis diagnosis. This has caused doctors to be mystified, and it has been a controversial issue in the establishment of treatment procedures [5].

In critically ill patients, particularly those with several comorbidities or prolonged ICU stay, the diagnosis of sepsis can occasionally be overstated, misleading the clinician into omitting a diagnosis due to the existence of multiple pathologies that mirror or overlap with sepsis.

Lower peripheral resistance with higher cardiac output and tachycardia are frequent hemodynamic consequences in the early stages of distributive shock, which can occur in allergy, pancreatitis, spinal injury, and other illnesses and also in septic shock. Later phases, which are similar to hypovolemic shock, are marked by increased vascular resistance, lower cardiac output, and cooler peripheral extremities with insufficient capillary filling. [6]. However, because clinical symptoms of confirmed or suspected sepsis can be varied and sometimes ambiguous, its diagnosis and management remain difficult [7].

Like the hemodynamic effects, laboratory findings are not specific for sepsis. An elevated white blood cell count with left shift may be caused by any kind of physiologic stress. Coagulopathy, fibrinogen, and the coagulation panel may show abnormalities in sepsis as well as other conditions that develop with a systemic inflammatory state, making distinction challenging. [6, 8]. Although leukopenia and thrombocytopenia are more suggestive of sepsis, they are insufficient to rule out the condition [6]. C-Reactive Protein has been the subject of many years of study to observe its dynamics in sepsis; however, it is elevated in any inflammatory condition and is not specific for sepsis. Procalcitonin (PCT) production is increased in response to bacterial infections and may quickly decrease during recovery. PCT gives valuable extra information that may be used to augment clinical and diagnostic criteria. Serum PCT is undetectable in healthy individuals in the absence of systemic inflammation. PCT is unable to discriminate between infectious and non-infectious systemic inflammatory response syndrome [7, 9].

Although lactic acid elevation and base excess/deficiency are frequently used in resuscitation, they do not give information on the cause of shock. Lactic acidosis may be caused by a variety of conditions, many of which are not infectious, the most common causes are circulatory failure and hypoxia [10].

Sepsis can mimic a variety of pathologies present in the ICU. Prompt diagnosis, volume resuscitation, and broad-spectrum antibiotic therapy initiated in the first hours after the onset of sepsis increase the patient’s chances of survival.

*Anaphylaxis* is the most severe allergic response; it affects many organ systems, is induced by a variety of triggers and conditions, and the patient can present with distributive shock symptoms -vasodilatation and hypotension. The key diagnosis is based on the patient’s history of allergies and possible exposure to triggers. Tryptase is a marker of mast cell activation that is evaluated at 30, 60, and 120 minutes after the beginning of an anaphylactic response [11].

In an emergency, *euglycemic diabetic ketoacidosis* with concomitant lactic acidosis is a typical presentation that might mask sepsis. High lactate levels in the absence of serum ketones aid in the diagnosis of sepsis [12].

In acute *pancreatitis*, SIRS is caused by the release of pancreatic enzymes. The presence of SIRS at admission may mislead the clinician. For a pancreatitis diagnosis, two of the three criteria must be met: abdominal pain, a lipase level three times normal, and ultrasound or abdominal CT characteristics consistent with pancreatitis. There are several severity scoring systems to aid in the prognosis of a patient’s clinical course, but many are time-consuming to calculate and frequently take more than 72 hours to become positive [13].

*Withdrawal state*- alcohol, benzodiazepine, and opioid withdrawal may result in SIRS. Li-Yuan Liu et al. described a 58-year-old man with a history of alcohol use who developed delirium as a result of sepsis-associated encephalopathy mimicking alcohol withdrawal delirium. Infections and alcohol withdrawal are two common causes of mental disorders. It’s difficult to discern between mental dysfunction due to alcoholism and sepsis when treating a patient with both. Prior episodes of alcohol withdrawal seizures or delirium, advanced age, detectable blood alcohol level on admission, excessive daily alcohol intake, impaired liver function, and male sex can all help lead the diagnosis. [14].

*Neuroleptic malignant syndrome* (NMS) is a possibly deadly neurologic condition caused by the use of neuroleptic drugs. Is often distinguished by a specific clinical condition of altered mental status, muscle rigidity, fever, and autonomic instability. Atypical cases of NMS might develop with the absence of muscle stiffness. Sepsis and NMS might overlap in the ICU, making diagnosis challenging. The key diagnosis of NMS is patient exposure to trigger drugs [15].

Despite the fact that sepsis is a well-studied pathology, with definitions continuously being adjusted and adapted to new findings, diagnosing sepsis may be sometimes challenging, even for the most experienced among us. As Ralph Green said, *“If you can make the diagnosis, the treatment is easy, and the damage can be reversed. But making the diagnosis is tricky.”*

Dubito ergo sum.

## References

[1] Niederman MS, Baron RM, Bouadma L, Calandra T, Daneman N, DeWaele J, Kollef MH, Lipman J, Nair GB (2021). Initial Antimicrobial Management of Sepsis. Crit Care.

[2] Chiu C, Legrand M (2021). Epidemiology of Sepsis and Septic Shock. Curr Opin Anaesthesiol.

[3] Singer M, Deutschman CS (2016). Seymour et al The Third International Consensus Definitions for Sepsis and Septic Shock (Sepsis-3). JAMA.

[4] Boushra MN, Miller SN, Koyfman A, Long B (2019). Consideration of Occult Infection and Sepsis Mimics in the Sick Patient Without an Apparent Infectious Source. Journal of Emergency Medicine.

[5] Dugar S, Choudhary C, Duggal A (2019). Sepsis and Septic Shock: Guideline-Based Management. Cleve Clin J Med.

[6] Long B, Koyfman A (2017). Clinical Mimics: An Emergency Medicine– Focused Review of Sepsis Mimics Journal of Emergency Medicine.

[7] Gregoriano C, Heilmann E, Molito A, Schuetz P (2020). Role of Procalcitonin Use in the Management of Sepsis. J Thorac Dis.

[8] Ding R, Wang Z, Lin Y, Liu B, Zhang Z, Ma X (2018). Comparison of a New Criteria for Sepsis-Induced Coagulopathy and International Society on Thrombosis and Haemostasis Disseminated Intravascular Coagulation Score in Critically Ill Patients with Sepsis 3.0: A Retrospective Study. Blood Coagulation & Fibrinolysis.

[9] Ryu J -A, Yang JH, Lee D, Park C-M, Suh GY, Jeon K, Cho J, Baek SY, Carriere KC (2015). Chung CR Clinical Usefulness of Procalcitonin and C-Reactive Protein as Outcome Predictors in Critically Ill Patients with Severe Sepsis and Septic Shock PLOS ONE.

[10] Nalos M, Robergs R (2019). Understanding Hyperlactatemia in Sepsis: Are We There Yet?. Am J Respir Crit Care Med.

[11] Bilò MB, Martini M, Tontini C, Corsi A, Antonicelli L (2020). Anaphylaxis. Eur Ann Allergy Clin Immunol.

[12] Nasa P, Chaudhary S, Shrivastava PK, Singh A (2021). Euglycemic Diabetic Ketoacidosis: A Missed Diagnosis. World J Diabetes.

[13] James TW, Crockett SD (2018). Management of Acute Pancreatitis in the First 72 Hours. Curr Opin Gastroenterol.

[14] (2022). Liu Delirium Due to Sepsis-Associated Encephalopathy Mimicking Alcohol Withdrawal Delirium Available online.

[15] Manabe S, Yanagi H, Ozawa H, Takagi A (2019). Neuroleptic Malignant Syndrome as Part of an Akinetic Crisis Associated with Sepsis in a Patient with Lewy Body Disease. BMJ Case Rep.

